# Effectiveness of CAD/CAM titanium fixed lingual retainer versus conventional stainless steel fixed retainer (randomized controlled clinical trial)

**DOI:** 10.1007/s00784-025-06418-x

**Published:** 2025-06-18

**Authors:** Nada E.A. Elhosseiny, Wessam M. Marzouk, Mostafa A. Tageldin

**Affiliations:** 1 Department of Orthodontics, Faculty of Dentistry, Alexandria University, Champolion St., Alexandria, Azarita, 21527 Alexandria Egypt; 2 Faculty of Dentistry, Alexandria University, Alexandria, Egypt; 3 Faculty of Dentistry, Alexandria University, Alexandria, Egypt

**Keywords:** CAD/CAM, Fixed retainers, Lingual retainer, Tooth irregularity, Intercanine width

## Abstract

**Background:**

Retention after orthodontic treatment is crucial to maintain aligned teeth and prevent relapse. Traditional fixed retainers, made from multistranded stainless steel wires, are effective but have drawbacks such as operator variability, long chair-time, and risk of biofilm accumulation. Recent advancements in computer-aided design/computer-aided manufacturing (CAD/CAM) technology proposed custom-designed fixed retainers as a modern solution.

**The aim of this trial:**

Primary aim was to evaluate the alignment stability of CAD/CAM grade 5 titanium (Ti5) versus stainless steel lingual fixed retainers. Secondary aim was to assess periodontal health over 6-months.

**Methods:**

A single-center, two-arm, parallel randomized controlled clinical trial with a 1:1 allocation ratio was carried out to address the aims of the study. Participants were randomly allocated into two equal groups: Group I received Ti5 CAD/CAM lingual retainers; Group II received conventional 8-strand braided stainless-steel fixed retainers. Both retainers were bonded directly on the six mandibular anterior teeth. Measurements were performed at 3 time points: immediately after retainer placement (T0), after 3 months (T3) and after 6 months (T6). Intercanine Width, Little’s Irregularity Index and tooth movements in buccolingual, mesiodistal, occluso-gingival and rotational directions were recorded for each tooth by superimposition of digital models. In addition, periodontal parameters (Plaque index, Gingival index, Probing depth and Bleeding on probing) were assessed.

**Results:**

Initially, 54 patients were screened for eligibility. A total of 36 patients were randomly assigned, completed follow-up, and were included in the analysis. The used test for comparison between variance were Chi-square, Welch’s t test and Greenhouse-Geisser test. Inter-canine width showed no statistically significant differences between the two groups at T0, T3, or T6 (*p* = 0.769, *p* = 0.440 and *p* = 0.827 respectively). Little’s Irregularity Index revealed no statistically significant difference between the two retainers at T0 (*p* = 0.123), T3(*p* = 0.562) and T6(*p* = 0.611). No significant changes in tooth positions were observed in the two groups. The mean change of Gingival index showed a significant decrease in CAD/CAM group compared to conventional at (T3 and T6 *p* = 0.001). The periodontal parameters were significantly better in the CAD/CAM retainers after 6-months.

**Conclusions:**

Ti5 CAD/CAM and conventional retainers are effective in maintaining intercanine width. Ti5 CAD/CAM retainers are capable of maintaining tooth position in three dimensional directions. Ti5 CAD/CAM retainers showed less deterioration of the periodontium in short-term observation.

## Background

Maintaining the stability of orthodontic treatment outcomes remains an important challenge in preventing relapse. Therefore, the retention phase is a critical step for each orthodontically treated case [1]. There is no universal consensus to favor a particular retention due to the lack of high-quality evidence regarding the type of retention to be followed or the material to be used in retention protocol [2]. The choice of retention protocol and appliance preference is largely determined by the orthodontist’s experience, patient’s expectations and the clinical circumstances. Retainers should be easily tolerated by the patient with little detrimental impact on speech, mastication, oral hygiene, comfort and overall oral tissue health [3].

The literature documents a wide range of fixed retainer (FR) protocols, highlighting significant variations in wire materials, bonding techniques, and the number of teeth involved [2]. FRs had demonstrated efficacy, reliability in short-term stability of teeth [4]. However, FRs can affect patient comfort and make oral hygiene maintenance more challenging [5]. A narrative review summarized randomized clinical trials evaluating the effectiveness of different retention appliances and concluded that bonded retainers are the most effective in stabilizing lower incisors. However, they can allow extraction spaces to reopen and make oral hygiene difficult, leading to increased plaque accumulation [6].

The CAD/CAM technology was recently introduced in the orthodontic field, especially for custom made orthodontic appliances such as clear aligners, customized labial or lingual systems and extended to orthodontic fixed retainers [7]. There are two types of manufacturing techniques: the additive and subtractive manufacturing:, The process of additive manufacturing, often referred to as 3D printing, includes adding successive layers of a specific material to build an object, while subtractive manufacturing or machining such as milling, turning or drilling uses a carefully planned tool movements to cut away material from a block material to form the desired object [8]. Advancements in CAD/CAM technology have led to the development of custom-made FRs, addressing the drawbacks of conventional retainers [9]. CAD/CAM systems consist of three main components: first, data acquisition unit that gathers information from the dental arch. This can be done directly using an intraoral scanner, creating a virtual model, or indirectly by scanning a stone model from a conventional impression. The second part involves software which designs a virtual model and calculates milling parameters. Finally, the computerized milling unit manufactures the design from a solid material block [10].

Titanium Grade 5 (Ti5), composed of the Ti–6Al–4 V alloy, is a preferred material due to its high mechanical strength. The addition of aluminum and vanadium enhances its corrosion resistance, making it superior to other titanium grades [11, 12]. CAD/CAM introduced new structural designs and materials tailored to digital fabrication [13]. An in vitro study by Roser et al. [14)] demonstrated that CAD/CAM and multistranded retainers differ in their restriction of tooth mobility. Nickel-titanium and Ti5 CAD/CAM retainers allowed greater tooth mobility compared to multistranded retainers [15]. Ti5 was the only material that did not fail during the aging process and demonstrated load capacity values comparable to those of the Twist flex retainer. Therefore, Ti5 CAD/CAM retainers may serve as a viable alternative. Ti5 CAD/CAM FRs emerge as a promising alternative to conventional FRs, offering enhanced precision, reduced chair-time, and less dependence on operator skill [16]. Moreover, they exhibit high predictability particularly in scenarios with limited bonding surfaces or anatomically challenging regions [17]. However, the clinical effectiveness of CAD/CAM FRs has not been extensively evaluated despite these potential benefits. The primary aim of the present study was to evaluate the alignment stability of CAD/CAM grade-5 titanium versus 8-strand braided stainless steel lingual fixed retainers and secondary aim was to assess periodontal health over 6-months.

## Methods

### Trial design

A single-center, two-arm, parallel randomized controlled clinical trial (RCT) with a 1:1 allocation ratio was carried out to address the aims of the study. Ethical approval number 0510 − 10/2022 was obtained from the Research Ethics Committee of the Faculty of Dentistry, Alexandria University (IRB: 00010556–IORG: 0008839). The trial was registered in the Pan African Clinical Trial registry (PACTR202310525946306) on October 12, 2023.

### Sample size calculation

The sample size was calculated using G*Power version 3.1.9.2 [18]. A mean difference in the ICW change of 0.5 mm was used as the mean difference for comparison between the two groups, and the standard deviation was set at 0.5 based on the study by Shim et al. [9]. Adopting a power of 80% (β = 0.20), and a level of significance of 5%, the minimum required sample size to detect standardized effect size (d) of 0.979 difference in the decrease of intercanine width was found to be 17 patients per group. The sample was increased to 18 patients per group after adjustment for dropouts [19, 20].

### Participants, settings and eligibility criteria

A total of 36 participants were included in the RCT, who had completed their active orthodontic treatment in the Department of Orthodontics clinic of the Faculty of Dentistry, Alexandria University.

### Inclusion criteria

Participants exhibiting a normal enamel structure in the mandibular anterior teeth. Compliance with the prescribed treatment regimen and the maintenance of adequate oral hygiene, as indicated by a score of less than 2 on the Simplified Oral Hygiene Index [21].

### Exclusion criteria

Individuals with a history of cleft lip or palate, or those diagnosed with a craniofacial syndrome [22].Participants with systemic diseases known to impact bone metabolism.

Patients who have undergone treatment with orthodontic lingual appliances, or those who have been re-treated orthodontically. The presence of active caries, restorations and fractures of anterior teeth. Periodontal disease affecting the anterior teeth.

### Randomization

The allocation list was generated using random allocation software (www.random.org) [23, 24]. Each allocation was identified by a code, which corresponded to the participant’s serial number in the study, along with the group names. These allocations were then sealed in sequentially numbered opaque envelopes by an assistant. The set of envelopes was entrusted to the senior supervisor. Whenever a new participant was enrolled for intervention, the supervisor provided the designated envelope to the operator. The participants in group I received 0.013 × 0.027-in customized Ti5 CAD/CAM retainer and group II received 0.011 × 0.027-in dead soft ribbon shaped 8-strand braided stainless-steel wire retainer (Bond.A.Braid, Reliance, Inc., USA).

### Intervention

All patients signed an informed consent after being informed about the purposes, retention benefits, risks and monitoring time of the study. Full mouth scaling and polishing were performed to all patients before retainer bonding followed by emphases on the proper oral hygiene instructions.

**Fig. 1:**
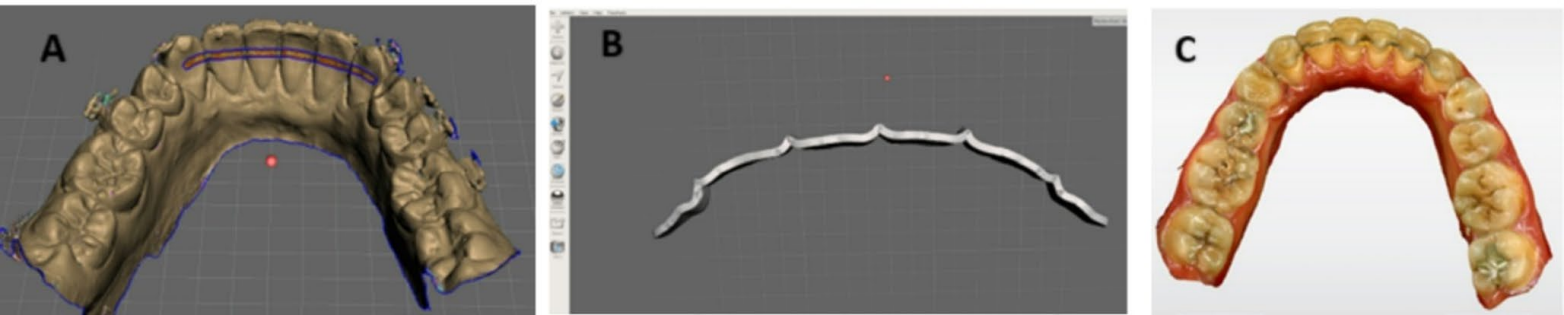
Designing of titanium FR on Meshmixer software; **A** Retainer design on imported STL file; **B** Extruded final design of FR from digital model (occlusal); **C** Intraoral scan of FR immediately after retainer bonding (T0)

Intra oral digital scanning were obtained for each patient using CEREC Omnicam intra-oral scanner (Sirona Dental Systems GmbH, Bensheim, Germany) before debonding of fixed appliances. In Group I intraoral scan was saved as a stereolithography (STL) file and imported into the Meshmixer software for further processing 3.5.474 (Autodesk Inc., CA, USA). The retainer was digitally designed on the lingual surfaces of the six mandibular anterior teeth by a trained operator. The digital model was aligned within the virtual workspace across three spatial planes, following pre-set software parameters. The scan voids were filled to generate a solid digital model using an appropriately sized drawing tool (Fig. 1), the retainer was designed with a thickness of 0.3 mm and a rectangular cross-section, positioned along the upper region of the middle third of the lingual surfaces, extending from one canine to the other. The retainer boundaries were then refined through a smoothing process.

The CAD/CAM retainer was fabricated using subtractive manufacturing, in which a CNC machine milled the structure from a Ti5 blank. CAM software converted the CAD model into a tool path by generating a series of commands that dictated the CNC machine’s sequencing, tool selection, and motion parameters. Tool positioning accuracy was maintained within 10 μm. Additionally, the CAM software incorporated compensation steps for cutter tool diameter, ensuring precise milling while preserving the integrity of the workpiece [25, 26]. The fabricated retainer surfaces were polished by using wheel brush and rubber cone burs except the fitting surface.

In group II, stainless-steel wire retainer was manually shaped and adapted directly on the lingual surface of patient’s mandibular six anterior teeth. All patients in both groups underwent the same bonding protocol by direct bonding technique by the same operator.

### Bonding protocol

**Fig. 2:**
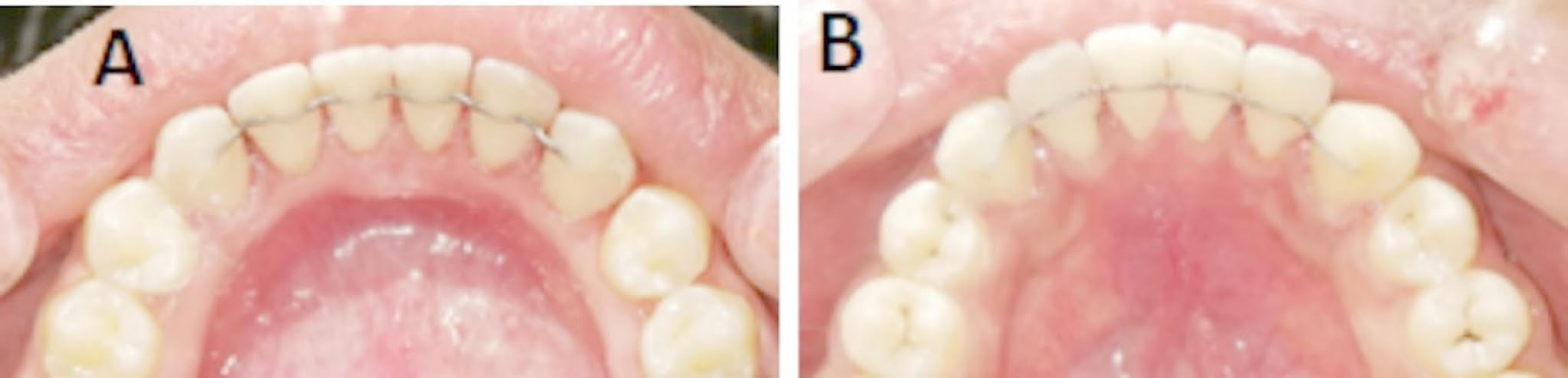
Clinical photos after retainer bonding; **A** CAD/CAM retainer; **B** Conventional StSt retainer

The mandibular lingual surfaces of incisors and canines were cleaned by non-fluoride polishing paste followed by sandblasting with aluminum oxide. Subsequently, the enamel surfaces were etched by 37% phosphoric acid gel (Meta Etchant, META BIOMED, Korea) for 30 s. Afterwards thorough rinsing and drying was performed then a layer of bonding material was applied (Assure Plus, Resilance, USA) and cured by iLed light cure (Guilin Woodpecker Medical Instrument Co., Ltd, Guangxi, China). Both retainers were fitted on the lingual surface of mandibular incisors using five dental floss threads. A mini-mold was used to apply a uniform layer of light-curing low-viscosity composite resin (Polofil, NHT flow, Germany) and each tooth was cured for 10 s. The bonded retainers were shown in Fig. 2.

### Patients instructions

Patients were advised to brush twice daily with a soft-bristled toothbrush and fluoride toothpaste, ensuring proper cleaning around the retainer wire. They were also instructed to use interdental brushes or floss threaders for cleaning under the wire and between teeth, along with an antibacterial mouthwash to minimize plaque accumulation. Regular dental visits and professional cleanings were recommended to assess oral health and retainer integrity. Additionally, patients were cautioned against biting hard foods or using their teeth as tools to prevent retainer damage [5].The patients were instructed to promptly report to the clinic in case of any retainer detachment or breakage.

Intra oral digital impressions were taken for all the patients at three time points:

T0: immediately after bonding of the FR.

T3: 3 months after T0.

T6: 6 months after T0.

## Outcomes

The following outcomes were assessed at T0, T3, and T6:

### Primary outcome

#### Dental alignment stability parameters

**Fig. 3:**
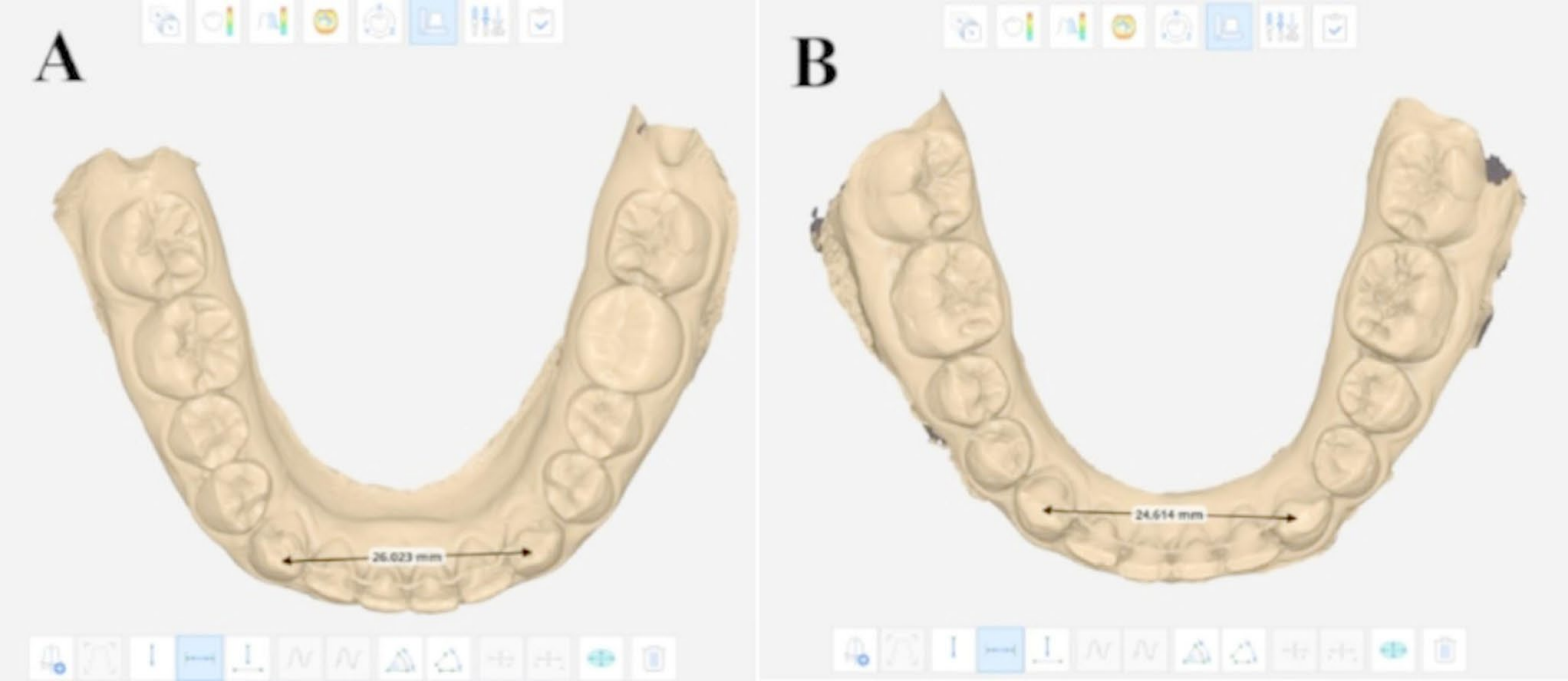
Intercanine Measurement on Medit link design;**A** ICW in at T0 CAD/CAM group; **B** ICW at T0 in Conventional group

**Fig. 4:**
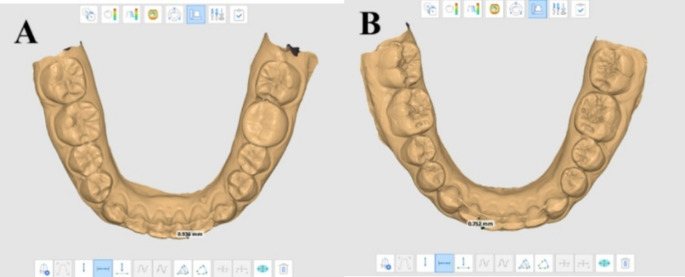
Irregualrity Index measurement on Medit link design; **A** LII at T1 in CAD/CAM group; **B** LII atT1 in Conventional group

The intercanine width (ICW) and Little’s irregularity index *(*LII*)* [27] measurements were assessed for each digital model using MEDIT Link 3.1.4 and MEDIT Design ^M^ 2.1.3 software. The ICW was defined as the linear measurements in millimeters (mm) from the cusp tip of one canine to the cusp tip of the other canine (Fig. 3). The LII value was measured by adding five distances between the anatomic contact areas from the mesial aspect of the right canine to the mesial aspect of the left canine in mm (Fig. 4). The mean and absolute change measurements were calculated and compared for the two groups.

### Digital superimposition

**Fig. 5:**
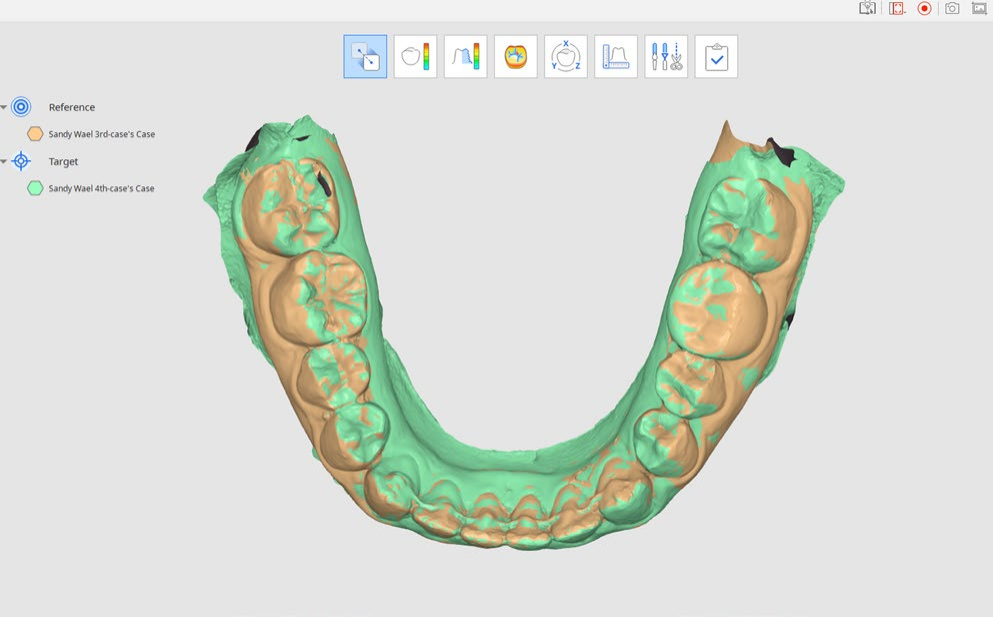
Digital models superimposition

A comparison of 3D surface models was accomplished by superimposing the two STL files on selecting three landmark points between the 1st and 2nd premolar, 2nd premolar and 1st molar and 1st molars and 2nd molar on the mucogingival line of each digital model according to Ioshida et al. protocol [28] (Fig. 5). Superimposition was performed on MEDIT Link 3.1.4 and MEDIT Design ^M^ 2.1.3 software along with OnDemand3D version1.0.

The buccal and lingual facial axis of the clinical crown (FACC) were identified three-dimensionally as lines passing through the most prominent parts of the buccal surfaces and their projections onto the lingual surfaces. The inclination of the FACC along the X- and Y-axes of an XYZ reference system was used to measure tooth movement in buccolingual, mesiodistal, and occluso-gingival directions across three planes of space [29].

#### Linear measurements assessment

##### Bucco-lingual movement

**Fig. 6:**
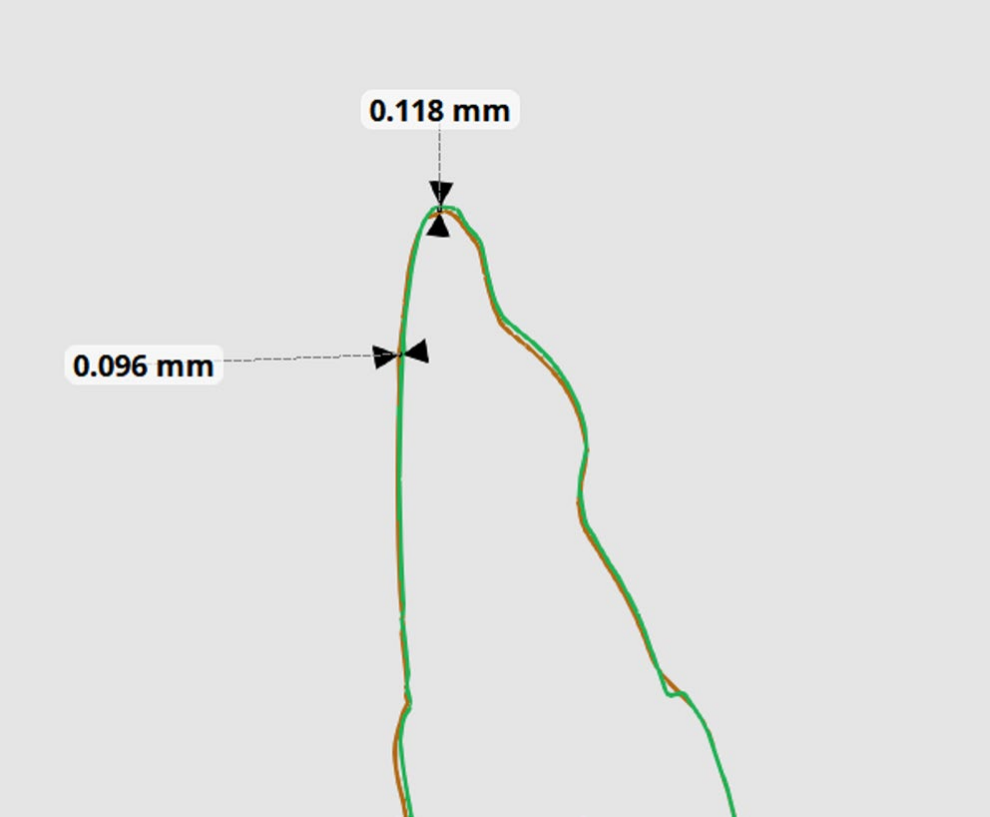
Buccolingual and Occluso-gingival movements measurements in proximal view (X-axis)

This was assessed by having a cross-sectional cut for each tooth on superimposed STL files and the linear distance was measured from the highest contour of the tooth to the line representing the long axis of the tooth when viewed from proximal aspect (Fig. 6).

##### Mesiodistal movement

**Fig. 7:**
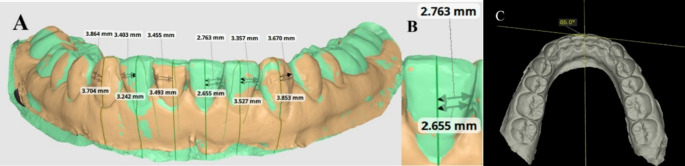
Mesiodital and Rotational movement; **A** Mesiodital measurement of each tooth after digital superimpostion from 3–3; **B** Difference in mesiodistal direction in a sectioned view; **C** Rotational angle in occlusal view

This was recorded from subtracting the linear distance from the disto-facial-incisal point angle to the line representing the long axis of each tooth at y-axis (Fig. 7).

##### Occluso-gingival movement

This vertical change was measured by subtracting the linear distance from the tip of the two incisal edges of a cross-sectional cut for each tooth on the superimposed STL files at x-axis (Fig. 6).

#### Angular measurement assessment

Rotation was calculated by measuring the angle between the tangent line to the highest labial contour of each tooth and a line bisecting the two lower centrals at the midline of lower arch for each STL file at z-axis for each STL file. (Fig. 7) Then, the two angles are subtracted to determine the amount of rotation for each tooth [30]. The discrepancy in linear and angular measurements were analyzed through statistical comparison for both groups.

### Secondary outcome

#### Periodontal assessments

The periodontal parameters as gingival index (GI), plaque index (PI), probing depth (PD) [31] and Bleeding on probing (BOP) were scored at T0, T3 and T6 for each group. PI and GI were assessed on the lingual surfaces for each tooth using a periodontal probe based on Löe scores [32]. PD was measured with a periodontal probe in mm at three locations (mesio-lingual, lingual, disto-lingual) for each tooth as the distance from gingival margin to the most apical part of the sulcus. BOP was examined and recorded either positive (bleeding observed) or negative (bleeding not observed) [33]. The average for the 6 mandibular anterior teeth were calculated for statistical analysis.

### Blinding

Patients were blinded regarding their assignment to study groups while, the operator could not be blinded due to the nature of the intervention. Additionally, blinding of the outcome assessor regarding stability (based on digital models) and periodontal clinical evaluation was not possible due to the evident differences between the retainers.

### Statistical analysis

Data analysis, patients’ records were made by the same operator to minimize observer and measurement bias. Data were collected and entered to the computer using SPSS (Statistical Package for Social Science) program for statistical analysis (version 25) [34]. Kolmogorov-Smirnov test of normality revealed no significance in the distribution of the variables. Data were described using mean, standard deviation, and 95% CI. Categorical variables were described using frequency and percentage. Comparisons were carried out between two studied independent normally distributed subgroups using independent sample t test [35].When Levene’s test for equality of variances was significant, Welch’s t-test was used [36, 37]. Comparisons were carried out between two studied dependent normally distributed subgroups using paired-samples t test [38]. Pearsons Chi-square test was used to test the association between qualitative variables [39]. Cochran’s Q test was used to verify whether the proportion of binary response is the same among different time intervals [40]. When Cochran’s Q test is significant, the pair-wise comparison was carried out using the Dunn-Sidak method [41]. Correction for *p* value was carried out using Bonferroni correction for multiple comparisons [42]. Repeated measures analysis of variance was used [43]. Model assumption was tested and found to be satisfactory with the exception of Mauchly’s test of sphericity [44], so Greenhouse-Geisser correction was used [45]. Statistical significance was tested at *p* value < 0.05 [46]. Percentage change was calculated as follows:

$$\:\varvec{P}\varvec{e}\varvec{r}\varvec{c}\varvec{e}\varvec{n}\varvec{t}\varvec{a}\varvec{g}\varvec{e}\:\varvec{c}\varvec{h}\varvec{a}\varvec{n}\varvec{g}\varvec{e}\:\left(\varvec{\%}\right)=\frac{\begin{array}{c}Measurement\:\left(\varvec{a}\varvec{f}\varvec{t}\varvec{e}\varvec{r}\right)\\\:-Meaurement\:\left(before\right)\end{array}}{\varvec{M}\varvec{e}\varvec{a}\varvec{u}\varvec{r}\varvec{e}\varvec{m}\varvec{e}\varvec{n}\varvec{t}\:\left(\varvec{b}\varvec{e}\varvec{f}\varvec{o}\varvec{r}\varvec{e}\right)}\times\:\:100$$

## Results

**Fig. 8:**
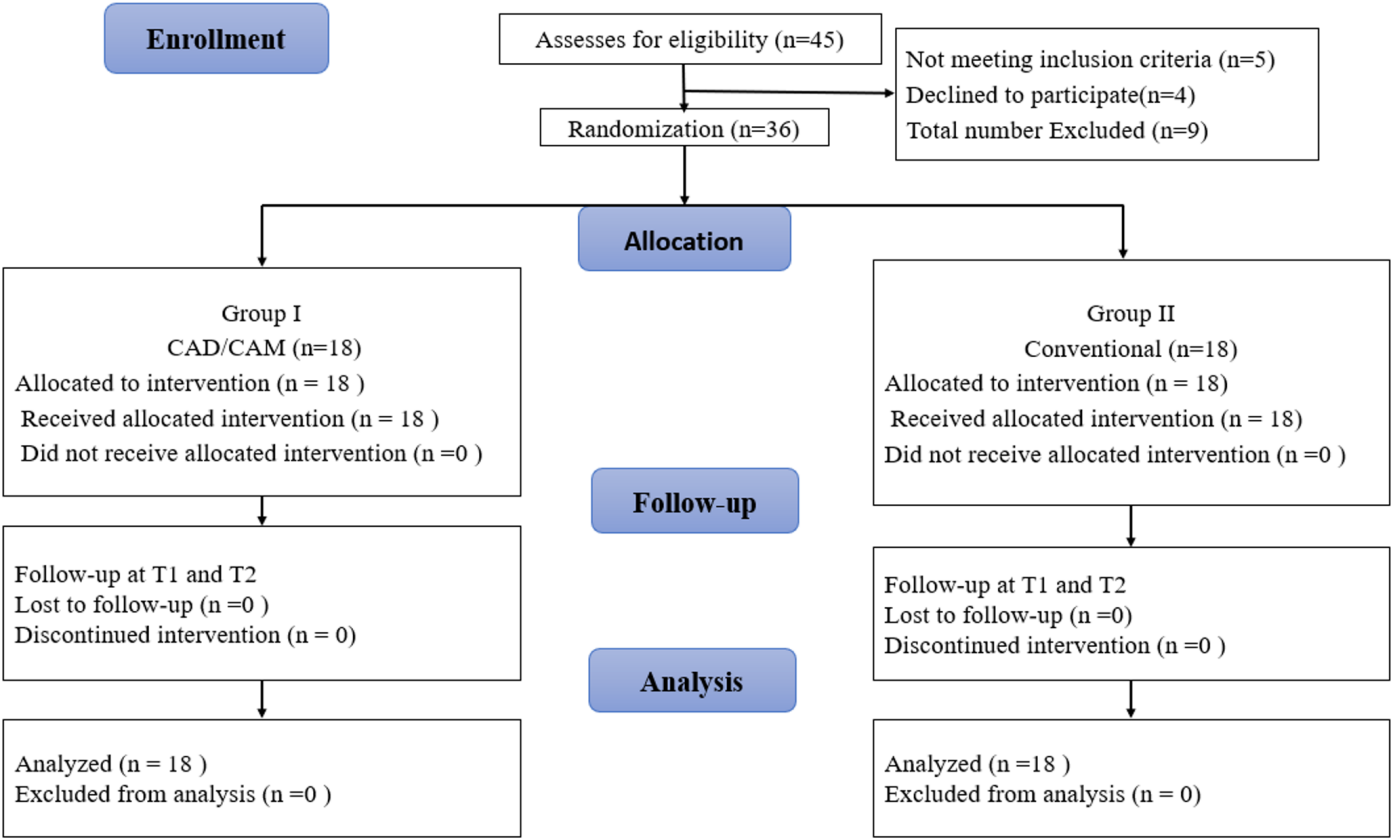
Patient flow diagram during the study

The demographic data for all patients are shown in Table 1. Forty-five individuals were evaluated for participation in this research. Five patients did not meet the inclusion criteria and four patients refused to participate in the trial. Therefore, a total of 36 individuals were randomized to two equal groups 18 each. The age and gender of the participants were homogenously distributed in both groups (*p* = 0.190 and *p* = 0.738 respectively). The flow of the patients through the trial were shown in Fig. 8.

**Table 1:** Sample distribution according to age and gender

Variable	CAD/CAM (*n* = 18)	Conventional (*n* = 18)	Test of significance *P*
**Age(years)** - Mean ± SD - 95% CI of the mean	19.890 ± 2.030 18.880–20.900	21.280 ± 3.880 19.350–23.210	t_(df=25.638)_ = 1.347 *p* = 0.190
**Gender** - **Male** n (%) = 17 (47.22%) - **Female** n (%) = 19(52.78%)	8 (47.06%) 10 (52.63%)	9 (52.94%) 9 (47.37%)	χ ^2^_(df=1)_ = 0.111 *p* = 0.738

n: Number of patients S.D.: Standard Deviation. CI: Confidence interval χ^2^: Pearson Chi-Square

### Stability measurements

The ICW and LII measurements was shown in Table 2. No statistically significant differences were observed between the two groups at T0, T3, or T6 (*p* = 0.769, *p* = 0.440, and *p* = 0.827, respectively). However, a significant intra-group reduction in ICW was noted in the CAD/CAM group across follow-ups (*p* = 0.004). The present findings suggested that both retainers maintained arch width and alignment similarly in inter-group comparisons.

**Table 2:** The irregularity index and Intercanine width in both groups during the study period

Variable		CAD/CAM (*n* = 18)	Conventional (*n* = 18)	*p*-value
**Intercanine Width (mm)**				
T0	mean ± SD 95% CI of mean	26.220 ± 1.420 25.520–26.930	26.090 ± 1.300 25.440–26.740	t_(df=34)_ = 0.296 *p* = 0.769
T3 T6	mean ± SD 95% CI of mean mean ± SD 95% CI of mean	26.070 ± 1.500 25.320–26.810 25.980 ± 1.530 25.210–26.740	25.560 ± 1.400 24.780–26.340 25.860 ± 1.570 25.080–26.640	t_(df=34)_ = 0.781 *p* = 0.440 t_(df=34)_ = 0.220 *p* = 0.827
Test of significance *p-value*		F_(GG)(df=1.410)_ = 8.518 *p =* 0.004*	F_(df=2)_ = 0.891 *p =* 0.419	
T3-T0	mean ± SD 95% CI of mean	-0.610 ± 0.950 -1.080 - -0.130	-1.420 ± 4.010 -3.410 -0.580	t_(df=34)_ = 0.831 *p* = 0.412
T6-T0	mean ± SD	-0.960 ± 1.230	-0.750 ± 5.970	t_(df=34)_ = 0.146
	95% CI of mean	-1.570 - -0.350	-3.72–2.220	*p* = 0.885
T6 -T3	mean ± SD 95% CI of mean	-0.360 ± 0.670 -0.690 - -0.020	0.640 ± 3.460 -1.080–2.360	t_(df=34)_ = 1.197 *p* = 0.240
**Irregularity Index (mm)**				
T0 T3	mean ± SD 95% CI of mean mean ± SD 95% CI of mean	0.129 ± 0.138 0.061–0.198 0.201 ± 0.116 0.143–0.259	0.070 ± 0.081 0.029–0.110 0.225 ± 0.130 0.161–0.290	t_(W)(df=27.596)_ = 1.591 *p* = 0.123 t_(df=34)_ = 0.586 *p* = 0.562
T6	mean ± SD	0.165 ± 0.165	0.191 ± 0.129	t_(df=34)_ = 0.514
	95% CI of mean	0.083–0.247	0.126–0.255	*p* = 0.611
Test of significance *p value*		F_(GG)(df=1.235)_ = 4.069 η_p_^2^ = 0.193 *p* = 0.049*	F_(GG)(df=1.451)_ = 8.504 η_p_^2^ = 0.333 *p* = 0.003*	
T3-T0	mean ± SD	0.070 ± 0.120	0.160 ± 0.180	t_(df=34)_ = 1.627
	95% CI of mean	0.010–0.130	0.070–0.250	*p* = 0.113
T6-T0	mean ± SD	0.040 ± 0.050	0.120 ± 0.110	t_(W)(df=23.861)_ = 3.053
	95% CI of mean	0.010–0.060	0.070–0.170	*p* = 0.005*
T6 -T3	mean ± SD	-0.040 ± 0.130	-0.003 ± 0.200	t_(W)(df=28.718)_ = 0.022
	95% CI of mean	-0.100–0.030	-0.140–0.070	*p* = 0.982

n: Number of patients CI: Confidence interval T0: Baseline; T3:3 months; T6:6 months. df = degree of freedom W: Welch’s t test GG = Greenhouse-Geisser η_p_^2^ = Partial eta squared *: Statistically significant (*p* < 0.05)

The LII measurements revealed no statistically significant difference between the two groups throughout the study period at T0 (*p* = 0.123), T3 (*p* = 0.562) and T6 (*p* = 0.611). Conversely, the mean change in LII at the 3-time evaluation points within each group was statistically significant in the two groups (*p* = 0.049 and *p* = 0.003 respectively). Additionally, the mean change of LII from T0 to T3 and T3 to T6 was insignificant between the two groups. On the other hand, the mean amount of irregularity decreased in CAD/CAM (0.040 ± 0.050) less than the conventional group (0.120 ± 0.110) with *p* value 0.050 after 6 months of follow-up.

The linear and angular measurement changes was presented in Table 3. The change for buccolingual measurements were insignificant at T3-T0 (*p* = 0.181) and T6-T0 (*p* = 0.909). Conversely, the CAD/CAM group exhibited a significant decrease in buccolingual movement from 0.130 to 0.078 mm over six months (*p* < 0.001). While the change was insignificantly decreased in conventional group throughout the different follow-up time from (0.106) to (0.080).

**Table 3:** Linear and angular measurements in both groups during the study period

Variance				CAD/CAM (*n* = 18)		Conventional (*n* = 18)		Test of significance *p*-value		
**Buccolingual (mm)**										
T3-T0 T6-T0		mean ± SD 95% CI of mean mean ± SD 95% CI of mean		0.130 ± 0.102 0.111–0.150 0.078 ± 0.075 0.064–0.093		0.106 ± 0.159 0.076–0.136 0.080 ± 0.084 0.064–0.095		t_(df=214)_ = 1.342 *p* = 0.181 t_(df=214)_ = 0.114 *p* = 0.909		
Paired t-test of significance *p-value*				t_(df=107)_ = 5.840 *P <* 0.001*		t_(df=107)_ = 1.606 *p* = 0.111				
**Mesiodistal (mm)**										
T3-T0 T6-T0		mean ± SD		0.208 ± 0.181		0.211 ± 0.193		t_(df=214)_ = 0.132 *p* = 0.895		
		95% CI of mean		0.174–0.242		0.175–0.248				
		mean ± SD		0.242 ± 0.236		0.239 ± 0.258		t_(df=214)_ = 0.105 *p* = 0.917		
		95% CI of mean		0.197–0.287		0.190–0.288				
Paired t-test of significance *p-value*				t_(df=107)_ = 1.283 *p* = 0.202		t_(df=107)_ = 0.948 *p* = 0.0345				
**Occluso-gingival (mm)**										
T3-T0		mean ± SD 95% CI of mean		0.153 ± 0.140 0.126–0.180		0.120 ± 0.247 0.073–0.167		t_(df=214)_ = 1.205 *p* = 0.229		
T6-T0		mean ± SD		0.087 ± 0.095		0.112 ± 0.248		t_(W)(df=138.104)_ = 0.979		
		95% CI of mean		0.069–0.105		0.065–0.159		*p* = 0.329		
Paired t-test of significance *p-value*				t_(df=107)_ = 4.315 *p <* 0.001*		t_(df=107)_ = 0.248 *p* = 0.804				
**Rotational angle (°)**										
T3-T0		mean ± SD 95% CI of mean		3.392 ± 2.495 2.916–3.868		4.585 ± 5.815 3.476–5.694		t_(W)(df=145.106)_ = 3.842 *p* = 0.052		
T6-T0		mean ± SD 95% CI of mean		3.115 ± 2.761 2.588–3.641		3.582 ± 2.298 3.244–4.021		t_(df=214)_ = 1.353 *p* = 0.178		
Paired t-test of significance *p-value*				t_(df=107)_ = 0.778 *p* = 0.438		t_(df=107)_ = 1.846 *p* = 0.068				

n: Number of patients. CI: Confidence interval T0: Baseline; T3:3 months; T6:6 months. W: Welch’s t test df = degree of freedom *: Statistically significant (*p* < 0.05)

Regarding the mesiodistal measurements, the changes were insignificant at inter- and intra-group measurements during evaluation time with *p*- value (0.895 and 0.917) and (0.202 and 0.034) respectively.

As for the change of the occluso-gingival tooth movement, all findings were insignificant except for the intragroup changes showed significant decrease in CAD/CAM group with *p* value *<* 0.001. Therefore, CAD/CAM retainers were capable to maintain the tooth position in all directions. All rotational measurements experienced insignificant change between the two retainers after 6 months.

### Periodontal measurements

The periodontal measurements are summarized in Tables 4 and 5. Plaque index (PI) showed no statistically significant differences in inter- or intra-group comparisons from T0 to T6, and the change remained insignificant throughout the follow-up period.

**Table 4:** Plaque index, gingival index and probing depth measurements in both groups during the study period

Variance		CAD/CAM (*n* = 18)	Conventional (*n* = 18)	*p*-value
**Plaque Index**				
T0 T3	mean ± SD 95% CI of mean mean ± SD 95% CI of mean	0.194 ± 0.319 0.035–0.353 0.259 ± 0.414 0.154–0.365	0.343 ± 0.350 0.170–0.517 0.231 ± 0.222 0.151–0.312	t_(df=34)_ = 1.340 *p* = 0.189 t_(df=34)_ = 0.259 *p* = 0.800
T6	mean ± SD	0.307 ± 0.325	0.620 ± 0.707	t_(W)(df=23.890)_ = 1.708
	95% CI of mean	0.194–0.418	0.476–0.972	*p* = 0.101
*Test of significance* *p-value*		F_(df=2)_ = 0.594 *p =* 0.558	F_(GG)(df=1.172)_ = 3.121 *p* = 0.087	
T3-T0 T6-T0 T6-T3	mean ± SD 95% CI of mean mean ± SD 95% CI of mean mean ± SD 95% CI of mean	− 11.960 ± 126.360 -144.567–120.647 23.343 ± 118.870 -100.582–147.268 15.589 ± 122.941 -78.912-110.090	-29.692 ± 66.458 -69.852–10.468 -29.692 ± 66.458 -69.852–10.468 53.408 ± 179.365 -67.091–173.908	t_(df=17)_ = 0.406 *p* = 0.689 t_(df=17)_ = 0.142 *p* = 0.889 t_(df=18)_ = 0.537 *p* = 0.598
**Gingival Index**				
T0	mean ± SD	1.139 ± 1.000	1.343 ± 0.949	t_(df=1214)_ = 1.536
	95% CI of mean	0.948–1.330	1.162–1.524	*p* = 0.126
T3	mean ± SD	0.676 ± 0.795	1.102 ± 1.032	t_(W)(df=200.971)_ = 3.398
	95% CI of mean	0.524–0.828	0.905–1.299	*p* = 0.001*
T6	mean ± SD	0.713 ± 0.832	1.259 ± 0.989	t_(df=214)_ = 4.391
	95% CI of mean	0.554–0.872	1.071–1.448	*P* < 0.001*
*Test of significance* *p-value*		F_(GG)(df=1.882)_ = 1.625 *p =* 0.201	F_(GG)(df=1.827)_ = 2.251 *p* = 0.113	
T3-T0	mean ± SD	-43.430 ± 38.060	-30.12 ± 63.460	t_(W)(df=137.059)_ = 1.603
	95% CI of mean	-52.43 - -34.42	-43.98 - -16.26	*p* = 0.111
T6-T0	mean ± SD	-44.840 ± 48.920	-21.29 ± 72.760	t_(W)(df=144.353)_ = 2.385
	95% CI of mean	-56.42 - -33.26	-37.17 - -5.40	*p* = 0.018*
T6-T3	mean ± SD	-7.100 ± 66.400	-1.21 ± 79.790	t_(W)(df=120.517)_ = 0.447
	95% CI of mean	-25.22–11.02	-20.38–17.96	*p* = 0.656
**Average Probing Depth (mm)**				
T0	mean ± SD	2.253 ± 0.175	2.299 ± 0.293	t_(W)(df=175.093)_ = 1.393
T3 T6	95% CI of mean mean ± SD 95% CI of mean	2.220–2.287 1.840 ± 0.210 1.800–1.880	2.243–2.355 2.001 ± 0.335 1.937–2.065	*p* = 0.165 t_(W)(df=179.975)_ = 4.229 *p* < 0.001*
	mean ± SD 95% CI of mean	1.478 ± 0.285 1.423–1.532	1.802 ± 0.343 1.737–1.868	t_(df=214)_ = 7.568 *p* < 0.001*
*Test of significance* *p-value*		F_(GG)(df=1.482)_ = 792.987 *p* < 0.001*	F_(GG)(df=1.730)_ = 101.317 *p* < 0.001*	
T3-T0	mean ± SD	-18.29 ± 7.070	-12.67 ± 12.440	t_(df=214)_ = 4.078
T6-T0 T6-T3	95% CI of mean mean ± SD	-19.64 - -16.94 -34.46 ± 10.890	-15.05 - -10.30 -20.67 ± 17.780	*p* < 0.001* t_(W)(df=177.401)_ = 6.872
	95% CI of mean	-36.54 - -32.38	-24.06 - -17.28	*p* < 0.001*
	mean ± SD	-19.99 ± 9.630	-8.52 ± 20.190	t_(W)(df=153.311)_ = 5.328
	95% CI of mean	-21.83 - -18.15	-12.37 - -4.67	*p* < 0.001*

n = number of patients. T0: Baseline; T3: 3 months; T6: 6 months. CI: Confidence interval

W: Welch test GG: Greenhouse-Geisser *: Statistically significant (*p* < 0.05)

**Table 5:** Bleeding on probing in both groups during the study period

Bleeding on probing	CADCAM (*n* = 108) %	Conventional (*n* = 108) %	Test of significance *p*-value
**T0** - Negative - Positive	67 (62.04%) 41 (37.96%)	63 (58.33%) 45 (41.67%)	c^2^_(df=1)_ = 0.309 *p* = 0.578
**T3** - Negative - Positive	80 (74.07%) 28 (25.93%)	56 (51.85%) 52 (48.15%)	c^2^_(df=1)_ = 11.435 *p* = 0.001*
**T6** - Negative - Positive	88 (81.48%) 20 (18.52%)	65 (60.19%) 43 (39.81%)	c^2^_(df=1)_ = 11.854 *p* = 0.001*
Cochran Q Test of significance. *p* < 0.001* *p-value*		*p =* 0.362	
**Post Hoc pairwise comparison for CAD/CAM Group**			
	**T0**	**T3**	**T6**
**T0**		*p* = 0.073	*p* = 0.001*
**T3**			*p* = 0.498

n: number of teeth T0: Baseline; T3: 3 months; T6: 6 months. *Statistically significant (*p* < 0.05) c^2^:Chi square

For the gingival index (GI), a significant reduction was observed in the CAD/CAM group compared to the conventional group at T3 and T6 (*p* = 0.001). While intra-group changes were not statistically significant in either group, the percentage reduction in GI from T0 to T6 was significantly greater in the CAD/CAM group (*p* = 0.018).

Probing depth (PD) measurements (31) demonstrated a significant decrease in the CAD/CAM group at T3 and T6 (*p* < 0.001). The mean PD in the CAD/CAM group was significantly lower than in conventional group at all time points: T0 (2.253 ± 0.175 mm), T3 (1.840 ± 0.210 mm), and T6 (1.478 ± 0.285 mm), with *p* < 0.001 at six months. Both groups exhibited significant intra-group reductions in PD over time (*p* < 0.001).

Bleeding on probing (BOP) findings in Table 5 indicated a significantly higher percentage of BOP in the conventional group (48.15%) compared to the CAD/CAM group (25.93%) at T3 (*p* = 0.001). After six months, BOP further decreased significantly in the CAD/CAM group (18.52%) compared to the conventional group (39.81%). Additionally, BOP continued to decline within the CAD/CAM group throughout the follow-up period (*p* < 0.001). The comparison of BOP within the CAD/CAM group showed a significant reduction at six months (*p* = 0.001) compared to three months (*p* = 0.073). However, the percentage change between T3 and T6 in the CAD/CAM group was not significant (*p* = 0.498).

These findings suggest that CAD/CAM retainers may contribute to improved periodontal health by reducing gingival inflammation, probing depth, and bleeding on probing over time, likely due to their smoother and more biocompatible surface, which minimizes plaque accumulation and bacterial adhesion.

## Discussion

The aim of the current RCT was to compare dental alignment stability and periodontal health between Ti5 CAD/CAM fixed retainers and standard conventional stainless-steel retainers.

Fabrication techniques in the present methodology favored milling over 3D printing for retainer fabrication, as milling is a subtractive manufacturing process that carves the retainer from a solid block of material. This approach ensures high precision, superior strength, and a smooth, polished surface that minimizes plaque accumulation [16, 47].

The present study observed variations in intercanine width (ICW) and Little’s Irregularity Index (LII) over time. The initial reduction in ICW after three months in both groups may reflect the natural settling of the teeth following fixed appliance removal, as occlusal forces begin to influence post-treatment alignment. The slight increase in ICW in the conventional group over the follow-up period could indicate minor relapse or arch expansion, possibly due to the increased flexibility of conventional retainers in the transverse dimension. Likewise, the initial increase in LII followed by a reduction indicates a temporary phase of minor irregularity post-debonding, which gradually stabilized with retainer use, this is consistent with the findings of Renkema et al. [48], who noted that an initial LII increase of less than 1 mm was not considered clinically significant.

Both retainers effectively maintained anterior alignment throughout the study; however, Ti5 CAD/CAM retainers may provide a slight advantage in long-term stability due to their precise adaptation and rigidity which likely contributed to enhanced resistance against transverse dimensional changes, thus promoting more stable alignment. A systematic review by Lambate et al. [49] found minimal differences in incisor cumulative displacement and apical labial changes between various CAD/CAM and conventional retainers. However, CAD/CAM retainers demonstrated a slight but non-clinically significant advantage in lower incisor inclination and a modest reduction in plaque index. Gingival index outcomes remained comparable between the two, reinforcing the viability of CAD/CAM retainers as an alternative to conventional options.

Another systematic review by Bardideh et al. [50] analyzed seven RCTs involving 601 participants. In the short term (less than six months), no significant differences were found in inter-canine distance or arch length between CAD/CAM and conventional fixed mandibular retainers, these results are in accordance with our findings, however, single-stranded stainless-steel retainers exhibited worse LII scores compared to Ni–Ti CAD/CAM retainers at 3 and 6 months, while multi-stranded stainless-steel retainers showed differences only at the 6-month evaluation. CAD/CAM retainers were associated with a lower plaque index than traditional retainers, though no significant differences were observed in the gingival index.

In vitro study conducted by Roser et al. [14] demonstrated that CAD/CAM and multistranded retainers vary in their ability to restrict tooth mobility. Nickel-titanium and Ti5 CAD/CAM retainers permitted greater tooth mobility compared to multistranded retainers. Conversely, CAD/CAM retainers composed of polyetheretherketone, zirconia (ZrO₂), and cobalt-chromium (CoCr) imposed significantly greater restrictions on tooth movement than multistranded retainers [14]. Additionally, Twistflex retainers remain the gold standard for long-term durability and Ti5 CAD/CAM retainers was the only material withstood the aging process without failure with load capacity comparable to Twistflex retainers. Therefore, Ti5 CAD/CAM retainers may represent a suitable alternative. We attribute these results to their precise fit on the teeth and smoother surface.

The present finding of the conventional retainer contradicts with Gunay and Oz [51] results, who reported an increase in irregularity in mandibular arch with both 0.0175-in 6-stranded stainless-steel wire and the 0.0195-in dead-soft coaxial wire. Similarly, Shim et al. [9] observed greater incisor irregularity with conventional stainless-steel Ortho-FlexTech fixed retainer wires in comparison with CAD/CAM retainers, this variation of results due to difference in techniques and types of retainer wire which might influence outcomes. Gera et al. [52] did not find significant differences in arch dimensions when comparing CAD/CAM Nitinol retainers with traditional 0.0215-inch six-stranded stainless steel fixed retainer. Shaughnessy et al. [53] also highlighted that the flexible spiral multistranded wires could contribute to such movements and suggested that a fully passive CAD/CAM fixed retainer might be less prone to causing these side effects.

The results showed no significant difference between the two groups regarding the linear and rotational changes over the six months. The comparable results of the individual tooth malposition between the two groups may indicate the efficiency of the two types of retainers to maintain teeth stability but, Ti5 CAD/CAM retainer provided an added advantage of positively impacting the periodontal health compared to conventional retainers. The findings of the current study suggest that CAD/CAM retainers may contribute to improved periodontal health by reducing gingival inflammation, probing depth, and bleeding on probing over time, likely due to their smoother and more biocompatible surface, which minimizes plaque accumulation and bacterial adhesion. This is consistent with Kartal et al. [33], who reported similar periodontal outcomes between CAD/CAM Nickel titanium Memotain^®^ and conventional five-stranded retainers manually bent retainer. Additionally, Knaup et al. [54] found that patients with CAD/CAM nitinol retainers had significantly lower plaque, gingival, and inflammatory indices, as well as less biofilm formation, compared to those with Twistflex retainers. Regarding the material, the Ti5 is known to be biocompatible, which was reflected in the current study results by offering some advantages in periodontal health. Such a promising impact on periodontal health is possibly due to the precise fit of the retainers with no room for food accumulation in addition to the inert biocompatible material properties.

Conversely, some studies have linked fixed lingual retainers to increased plaque accumulation and gingival inflammation. Wagner et al. [55] and Levin et al. [56] reported that fixed retainers were associated with increased gingival recession, plaque retention, and bleeding on probing. Also, Chapple [57] noted a connection between fixed retainers and the development of periodontal disease and attachment loss. These effects are likely due to the biofilm-promoting nature of multistranded fixed retainers. This is consistent with other studies [2, 58] which reported increases in probing depth, bleeding tendency, and tissue irritation associated with fixed retainers.

All reported findings indicate that Ti5 CAD/CAM retainers offer advantages in terms of both design and material, their durability and failure rates may vary based on factors such as bonding technique, retainer design, and individual patient characteristics. CAD/CAM technology enables the fabrication of precise, custom-designed retainers, ensuring an optimal fit, minimizing human error, and maintaining consistent quality. Additionally, Ti5 CAD/CAM fixed retainers feature smooth, polished surfaces that reduce biofilm accumulation, enhancing oral hygiene and promoting periodontal health. Also effective in preserving tooth position across multiple directions, providing superior alignment stability.

Emerging evidence supports the potential of metal-free retainers with various bonding protocols. The study by Scribante et al. [59] have shown promising results for metal-free retainers. Future research should compare these metal-free options with Ti5 CAD/CAM metal retainers using conventional bonding protocols to assess their long-term clinical efficacy.

### Limitations

Patients blinding helped to minimize bias and establish a clearer relationship between the intervention and outcomes, However, the absence of operator blinding may have introduced potential bias in the assessment process. Additionally, the short-term evaluation period limits the ability to assess long-term retention efficacy, tooth alignment stability, and the impact of CAD/CAM retainers on periodontal health.

Future long-term studies may provide more comprehensive insights. Furthermore, the inclusion of both extraction and non-extraction cases presents a limitation as stability may differ between these groups.

Moreover, the cost-effectiveness of CAD/CAM and conventional retainers could not be assessed due to the limited sample size and short evaluation period. Differences in clinical time application between the two types of retainers may also impact overall cost considerations.

Lastly, the lack of clinical trials directly comparing CAD/CAM retainers with other retention methods highlights the need for further research in this area. Expanding studies to include larger sample sizes and extended follow-up durations will be essential for confirming the long-term benefits and limitations of CAD/CAM retainers.

## Conclusion

Considering a relatively short observation period of six months, the findings of the present study provide the following conclusions:

1. Both Ti5 CAD/CAM retainers and conventional stainless-steel retainers are effective in maintaining intercanine width.
2. Ti5 CAD/CAM retainers are capable of maintaining tooth position in 3D directions.
3. Ti5 CAD/CAM retainers showed a tendency towards less deterioration of the periodontium.

## Data Availability

The datasets analyzed in the current research are available at synapse.org, under the title: Fixed retainers, 10.7303/syn64829606.
